# Patricide and overkill: a review of the literature and case report of a murder with Capgras delusion

**DOI:** 10.1007/s12024-020-00314-4

**Published:** 2020-09-18

**Authors:** Silvia Trotta, Gabriele Mandarelli, Davide Ferorelli, Biagio Solarino

**Affiliations:** 1 Institute of Legal Medicine, Department of Interdisciplinary Medicine (DIM), Policlinico di Bari Hospital, University of Bari, Piazza G. Cesare 11, 70124 Bari, Italy; 2 Section of Criminology and Forensic Psychiatry, Department of Interdisciplinary Medicine (DIM), University of Bari, Piazza G. Cesare 11, 70124 Bari, Italy

**Keywords:** Patricide, Capgras syndrome, Overkill, Autopsy, Homicidal asphyxia

## Abstract

Despite being an infrequent crime, parental homicide has been associated with schizophrenia spectrum disorders in adult perpetrators and a history of child abuse and family violence in adolescent perpetrators. Among severe psychiatric disorders there is initial evidence that delusional misidentification might also play a role in parricide. Parricides are often committed with undue violence and may result in overkill. The authors present the case of an adult male affected by schizoaffective disorder and Capgras syndrome who committed patricide. Forensic pathologists classify such cases as overkill by multiple fatal means comprising stabbing, blunt trauma and choking. Accurate crime scene investigations coupled with psychiatric examinations of perpetrator allow reconstruction of the murder stages. This overkill case is discussed in the context of a broad review of the literature.

## Introduction

Parricide is a word of Latin origin referring to the killing of a close relative. In Roman times this kind of homicide was punished more severely than any other kind of homicide, with the so-called *poena cullei* (“penalty of the sack”), a form of punishment consisting of sewing up the murderer in a leather sack together with an assortment of live animals including a dog, snake, monkey, and a chicken or rooster, and then throwing the sack into water [1]. Nowadays, the term parricide is generally used to identify offspring-perpetrated homicides, which can involve juveniles or adults as perpetrators, and biological, adoptive or step parents as victims [2, 3]. Depending on which parent is the victim, different definitions are used: patricide refers to killing one’s father, matricide to killing one’s mother, and double parricide is defined as killing both parents [2, 4].

Parental homicide is an infrequent event accounting for only 2–5% of all homicides, depending on the country examined [5, 6]. Given the rarity of such crimes, most of the literature on parricide is outdated and consists of anecdotal case reports and small-cohort studies [7–10]. The limited existing evidence about the prevalence of parricide comes from small studies that yielded inconclusive results, patricide being considered the most frequent [5, 9], whereas double parricide is extremely rare [9]. Indeed, there is a general consensus that parricide is to be regarded as a crime perpetrated by males, while female parricides are rare [2, 11–13]. Criminological studies reported that victims are often described as domineering and punitive by the offenders, whose dependent personality traits have also been described [14, 15].

Forensic experts and mental health professionals have recognized that the perpetrators usually fall into three categories: abused or mistreated children, mentally ill offspring, and children with antisocial traits [2, 13, 16]. Studies on juvenile samples suggest that they mainly fall into the category of abused or mistreated children and that the criminal act is a response to long-standing abuse; on the other hand, in several cases of adult-perpetrated parricides, the offender seems to be affected by mental illness [2, 5, 12, 13, 16], in particular female-perpetrated parental murders being frequently associated with schizophrenia or depression [17–19]. The murder in most cases is committed in the house where perpetrators reside with the victim [9, 11, 12]. It has been observed that sons/daughters who kill their parents frequently use painful methods and excessive violence, sometimes employing multiple fatal methods, thus resulting in overkill [9, 16].

We report here a case of overkill patricide perpetrated by an adult male affected by a schizoaffective disorder and Capgras delusion, who killed his father using multiple fatal means; we also analyze the case in the light of the existing literature on parricide and overkill. Capgras syndrome is a type of delusional misidentification syndrome in which the subject holds that a well-known person has been replaced by an identical or very similar impostor [20]. Schizophrenia and other psychoses have been associated with increased risk of committing homicide [21] and there is initial evidence suggesting a possible association between parricide and delusional misidentification syndromes [22]. This case adds information to the limited literature on misidentification syndromes, and overkill homicide.

## Case report

An 83-year-old man, killed by his son, was found dead in the dining room of his apartment, with plenty of blood staining the surrounding floor and the walls. Following the murder, the offender phoned his sister and brother-in-law to reassure them that “everything had been fixed”. He then rang the neighbor’s doorbell, but they did not open the door, so he remained in a state of perplexity on their stairs with a tattered towel in his hands.

**Fig. 1:**
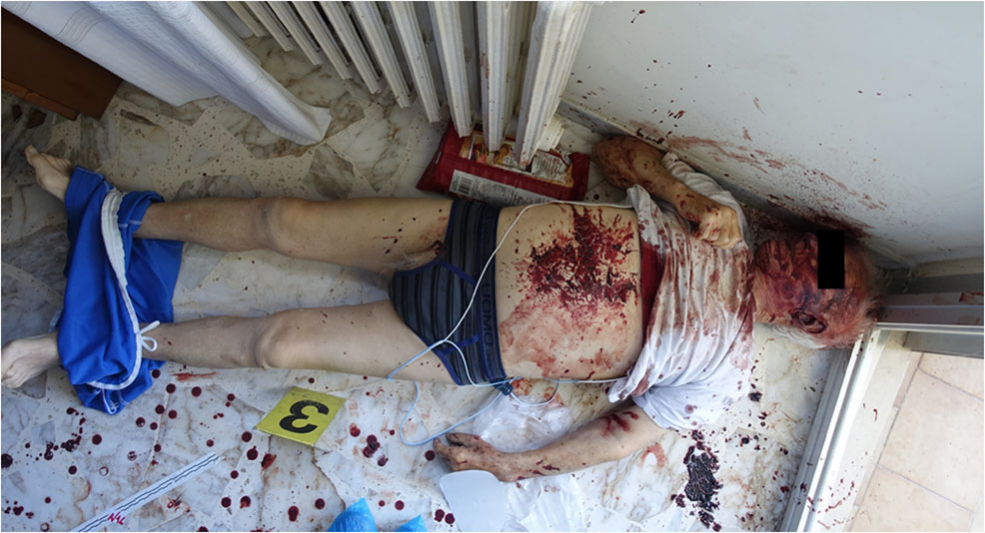
Victim lying on the floor with his head adjacent to a broken marble baseboard, holding a bread knife in his right hand

The victim was lying supine on the floor with his head adjacent to a broken marble baseboard. He was wearing a white t-shirt that had been raised above the chest, a pair of male underpants, and a pair of shorts that had been lowered to the ankles. He was holding a bread knife loosely in his right hand (Fig. 1); the blade measured 20 cm in length and 2.5 cm in width. The man’s face, abdomen and arms were covered with blood, and multiple buttonhole-shaped skin lesions were evident on the abdomen. No traces of blood were observed on the man’s legs or on his back. Two crime scene investigations were undertaken; before and after performing the forensic autopsy required by the public prosecutor.

### Autopsy

**Fig. 2:**
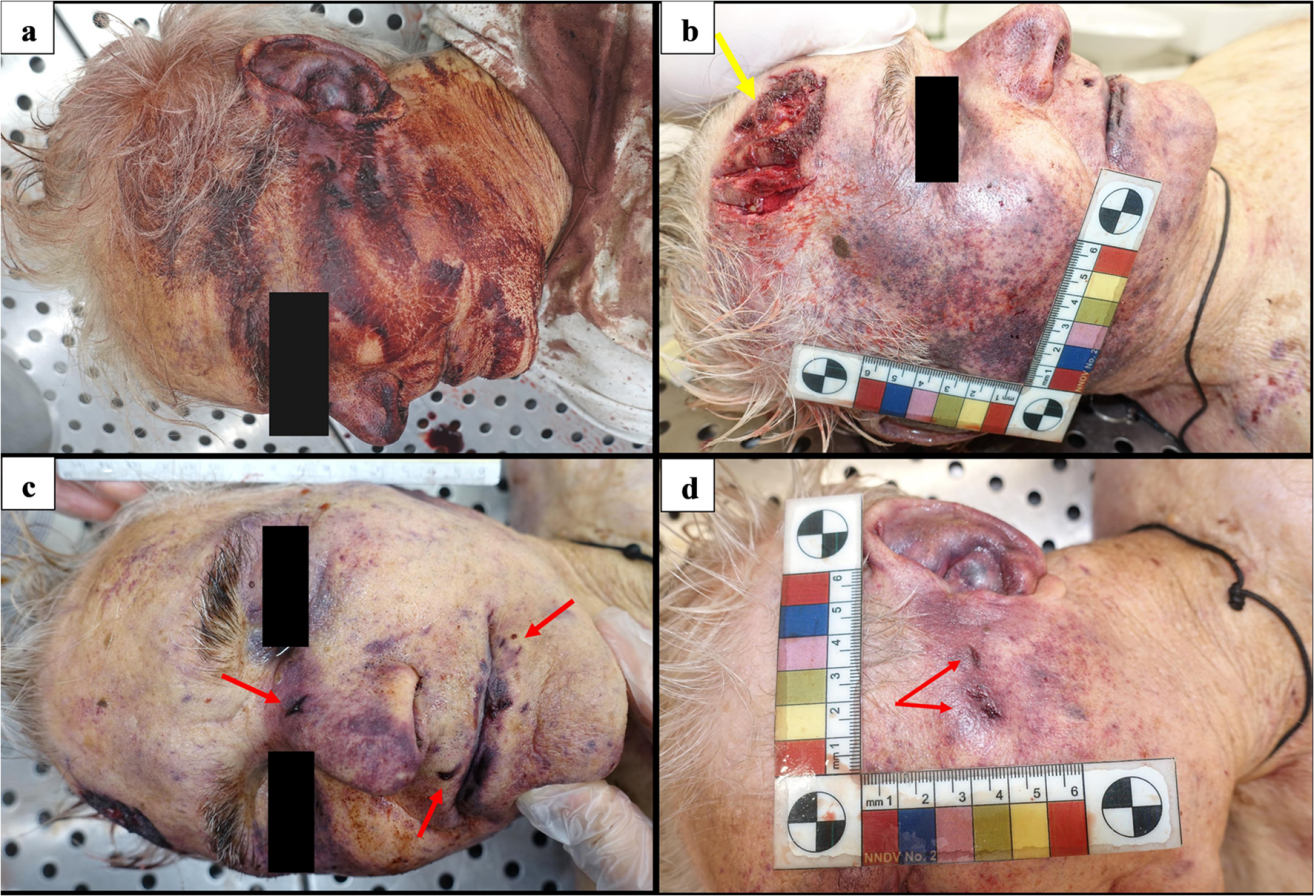
Details of the victim’s face. **a** Fingerlike imprints of dried blood; and on the left arm. **b** Lacerated wounds with jagged margins (yellow arrow) on the right frontoparietal region and contusion of the right side of the face. **c** Confluent hemorrhage of the eyelids and contusion of the nose; fingernail abrasions on the nose and around the mouth (red arrows). **d** Contusion of the left side of the face with fingernail abrasions (red arrows)

**Fig. 3:**
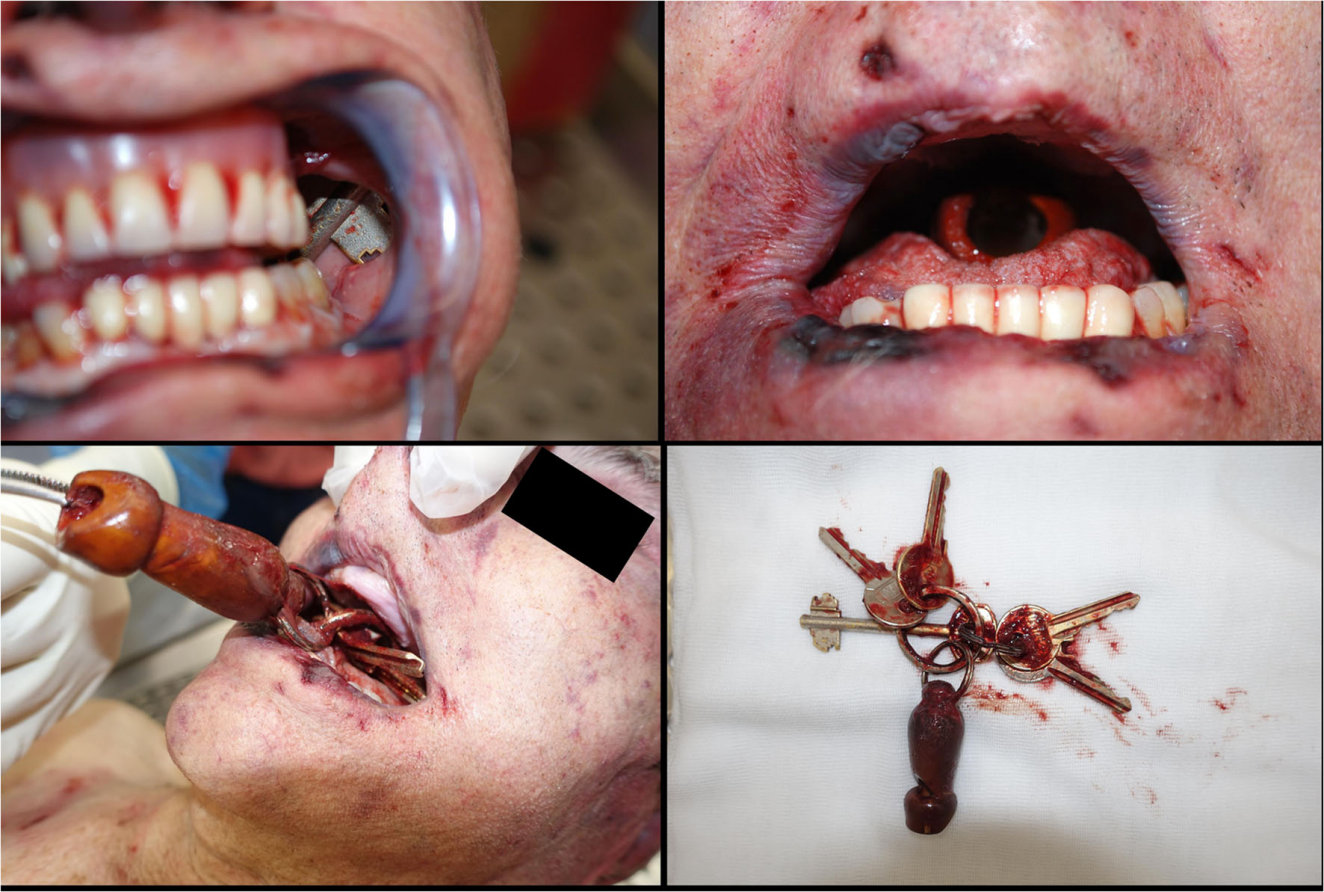
Set of keys with keychain in the oral cavity

**Fig. 4:**
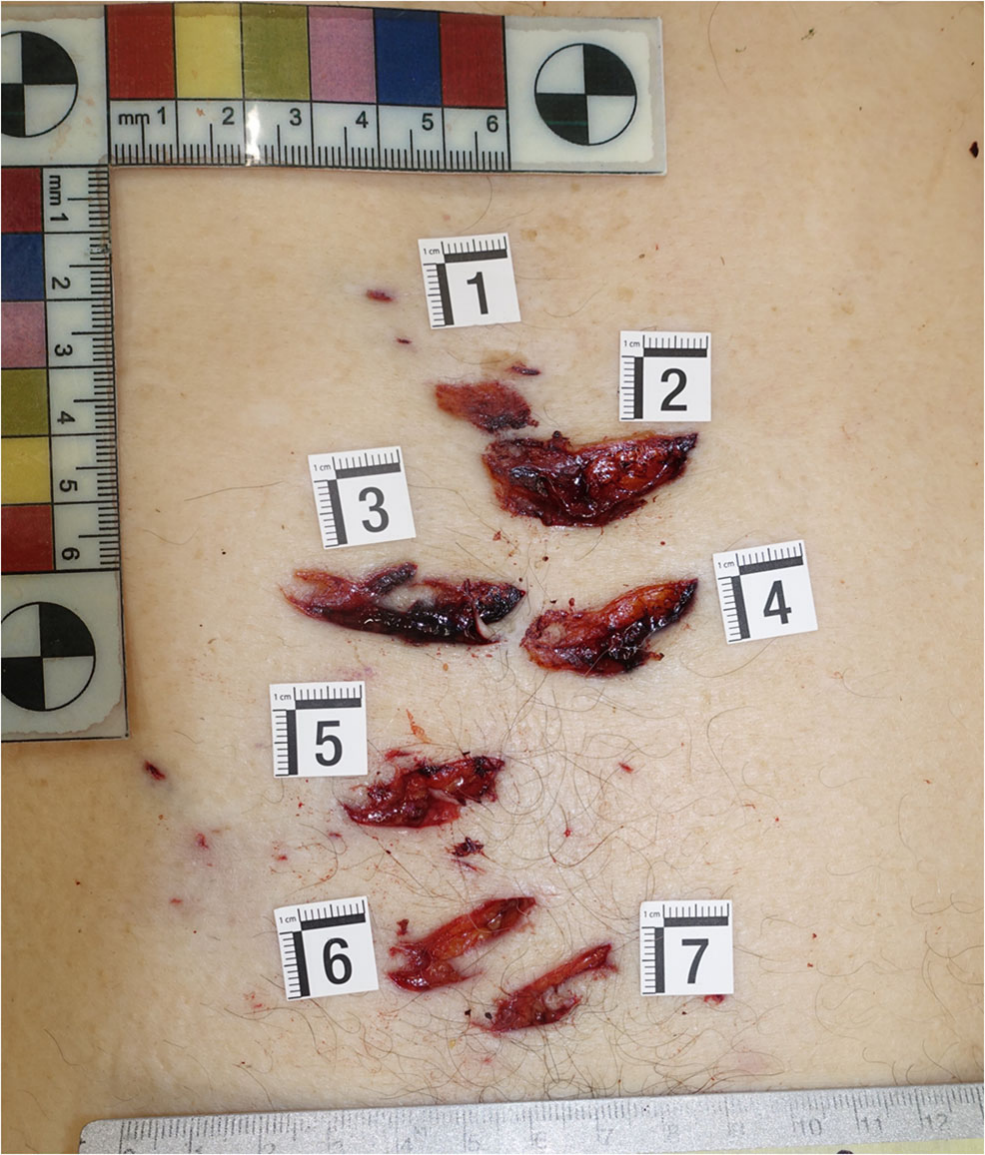
Skin lesions showing a single pointed end and a unilateral “fish tail” split

The external examination of the body revealed fingerlike imprints of dried blood on the left side of the victim’s face (Fig. 2a) and on the left arm. The scalp was widely contused, and a series of lacerated wounds with jagged margins was observed in an area measuring 6 × 4.8 cm on the right frontoparietal region (Fig. 2b). Confluent hemorrhage of the eyelids as well as contusion of the nose, and the right and left side of the face, were also detected (Fig. 2b, c). Numerous small abrasions, possibly reflecting injuries caused by fingernails, were described near the right eye, at the root of the nose, on the left cheek, around the mouth, and on the lower lip (Fig. 2c, d); such injuries were also seen on the neck and the upper part of the chest, surrounded by areas of slight contusion. Inspection of the oral cavity revealed the presence of a set of five metal keys with a whistle-like keychain, which were gently removed (Fig. 3). In the epigastric region, in an area measuring 12 × 9 cm, there were seven skin lesions showing a single pointed end and a unilateral “fish tail” split, consistent with stab wounds (Fig. 4). These injuries, numbered from 1 to 7, were of different sizes, the largest being those labelled from 2 to 4. Several bruises were observed on the back of the trunk and on the upper limbs.

**Fig. 5:**
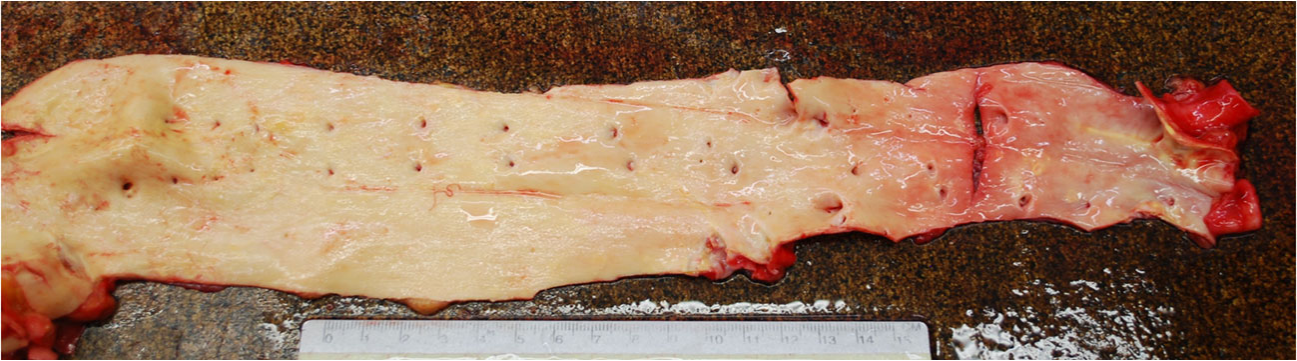
Injury of the abdominal aorta

At autopsy, a hemorrhagic infiltrate of the scalp and temporalis muscles was found, in the absence of skull fractures; a slight subarachnoid hemorrhage in the right temporal lobe was also observed. Hemorrhagic infiltration of the masseter and pterygoid muscles was described bilaterally. The tongue presented superficial blood infiltrates which were also detectable in the palatine region, along with a small laceration of the mucosa, and in the hypopharynx. Blood was seen in the trachea and the bronchial tree. The seven stab wounds showed trajectories which can be summarized as follows:

- 1 → superficial subcutaneous tissue;
- 2–3 → subcutaneous adipose tissue → muscles of the abdominal wall → anterior gastric wall → posterior gastric wall;
- 4 → subcutaneous adipose tissue;
- 5 → subcutaneous adipose tissue → muscles of the abdominal wall → greater omentum → mesenteric root;
- 6–7 → subcutaneous adipose tissue → muscles of the abdominal wall → greater omentum → mesenteric root → aorta (Fig. 5).

Hemorrhage infiltrates were revealed on the greater omentum and the mesentery. The volume of the hemoperitoneum was nearly 1.5 l and we observed a retroperitoneal hematoma that involved an area comprised of the left kidney fibrous and adipose tissues, the psoas muscles, and the surrounding bladder space. No skeletal fractures were observed. Toxicological analyses were negative.

### Psychiatric history of the offender

The perpetrator was the decedent’s 45-year-old unmarried son, who lived with his father. He was the third and youngest son, and no family history of psychiatric disorders was found. A regular psychosocial development was reported, and his personality was described as lively. He had graduated from high school despite failing 2 years. Thereafter, he had had occasional jobs before finding a permanent job in a shoe factory.

The man had been unemployed for 19 years before the crime, when he underwent his first psychiatric admission due to acute delusions; he was then diagnosed with a bipolar disorder. An initial unspecified psychopharmacological therapy was introduced, and the case notes analysis revealed good treatment adherence and a diagnostic change to a delusional disorder. Following a readmission 12 years prior to the crime, he was diagnosed with “depression and personality disorder”. At the time of the crime he was supposed to be taking prescribed quetiapine and lithium carbonate.

No significant changes were reported in the medical records until the day before the murder, when a request for a medical examination was made by the offender’s father, apparently due to treatment non-adherence. After the homicide, the forensic psychiatric expert examination yielded a diagnosis of a schizoaffective disorder with acute paranoid delusion. The expert concluded that the offender should be held criminally irresponsible due to acute delusion that motivated an uncontrollable homicidal behavior. The offender affirmed that he killed his father because he had been replaced by an impostor that threatened both his father’s and his own life. He also reported that at times he perceived the impostor as an inanimate object, such as a “sack of potatoes”.

## Discussion

A history of psychosis is frequent in parricide offenders, who often perceive their father as threatening due to delusions involving the parental figure [23, 24]. The killer usually blames the external situation for his internal tension and holds the victim responsible for his act [8]. A review of the studies carried out on parricides shows that adult male perpetrators often present the following common traits: they are frequently unmarried, unemployed and living with their parents at the time of the crime; murders usually take place in the victim’s house [2–4, 9–13, 15, 16, 23–25].

It has been observed that three circumstances are mainly associated with murder, namely a history of child abuse, serious mental illness, and financial issues [13, 16]. Thus, different factors influence this extreme criminal behavior, including anamnestic and clinical factors, as well as psychosocial stress.

Child abuse is one of the main factors associated with juvenile-perpetrated patricides [2, 5, 12]. Several studies have observed that adolescent parricides are more likely to have been repeatedly physically or sexually abused by their victim, to have witnessed conjugal violence or to have a neglectful father [5, 12, 25, 26]. Such circumstances could represent triggers for committing patricide [5].

Among clinical factors, an antisocial personality is one of the main risk factors for parricides among juveniles and it is often related with familicides involving parents and other kin [3, 27]. It has been observed that children who have grown up in dysfunctional families are at greater risk to develop a conduct disorder, which is a precursor of an antisocial personality disorder [2, 28–32].

Psychosis has also been associated with juvenile parricide in a minority of cases, whereas it is the most common psychiatric diagnosis in adult perpetrators [3, 5, 6, 10]. It is noteworthy that most of the cohort studies on parricides were carried out on inpatients of psychiatric hospitals [5, 10, 15, 16, 23, 24, 33], thus determining a possible bias of the data obtained. In these studies, a diagnosis of a chronic mental disorder and a history of inpatient treatment prior to parental homicide have often been highlighted [5, 10, 16, 24]. Although schizophrenia is regarded as the most common diagnosis in parricide offenders [5, 10, 15, 16, 24, 33], other psychiatric conditions such as depression [16, 30], schizoaffective disorder [5], and personality disorders [5, 10, 16, 33], have been reported. Epilepsy [10], perceptual abnormalities and misidentification syndromes [24] have also been described.

Despite showing a high prevalence of psychiatric disorders in parricide offenders, including psychotic disorders, major depression, bipolar disorder, and antisocial personality disorder, the few retrospective cohort studies on parricides were carried out on samples drawn from coroners’ files and forensic medical institutions [9, 13], and the only one based on forensic autopsy reports [3] shows that in a high percentage of cases the murderers have no mental disorder and the reason for parricide is often unknown or related to financial issues or to a history of domestic violence.

Such differences also emerge from the comparison of a double case report on double parricide [34], which outlines the close relation between schizophrenia with delusions and parental homicide, and a single case of patricide due to economic issues [4].

Overall, there is a strong consensus that considers psychosis with delusions or hallucinations as the main motivating factor for parricides.

Data regarding the association between alcohol or substance abuse and parricide are inconsistent, as some studies reported a significant association [9, 10, 33], while others did not [2, 23, 24]. Despite the fact that most individuals suffering from psychoses do not kill their parents [35], those who commit parricide often present treatment non-adherence or have been recently threatened with enforced hospitalization or enforced medication by their victim [3, 5, 24, 33, 35].

One more aspect which has been investigated in parricides is the weapon choice made by the offender. Globally, the choice of the weapon used to carry out a homicide depends on many variables, such as the context in which the murder takes place and the social availability of the instrument used [3, 36]. Thus, homicide by firearms is more frequent in the USA and Italy, as a result of less restrictive gun laws and homicides involving organized crime, respectively [36–38]. For parricide, firearms are quoted as the main weapon used in the studies carried out in the USA [39]. Some authors stated that fathers are more likely to be killed by firearms compared to mothers, who mainly tend to be slain with sharp instruments [2, 39]. It has also been observed that the methods used for killing differ with the age of the offenders: juveniles are significantly more likely to choose firearms, whereas adults tend to use knives, blunt objects, personal weapons or asphyxiation, regardless of the victim’s sex [5, 39, 40]. On the contrary, most studies carried out on parricides highlight that parental homicides are often perpetrated using blunt or sharp instruments [3, 9, 10, 13, 15, 24, 33]. This might be due to the impulsiveness of such crimes, which are seldom premeditated, therefore the choice often falls on the most accessible instrument or weapon [2, 3, 37]. It has been postulated that the relationship between the offender and victim is strongly associated with the type of weapon chosen: blunt instruments seem to be used in a high proportion of homicides involving family members and to be strongly associated with an outburst of emotions in offenders [41], whereas in sharp-force homicides, the higher the number of injuries sustained by the victim, the closer the victim-offender relationship [41, 42]. Forensic psychiatrists also suggest that the type of mental disorder of the perpetrator may influence the choice of the weapon [36, 37], with depatterning of behavior, common in schizophrenics, leading to a random choice at the crime scene, whereas delirium or hallucinations may explain the choice of unusual or atypical weapons [36]. Indeed, some associations between the mental disorder of the offender and the weapon used have been described in the literature, the most common being schizophrenia/delusional disorder with the use of sharp instruments [36, 43], often correlated with multiple strikes on the torso [36] and mood disorders with strangling, asphyxiation, suffocation, and drowning [43]. A personality disorder, alcohol dependence and organic disorders are often correlated with the use of blunt instruments [43].

Given both the particular nexus of biopsychosocial factors connecting children with their parents, and the psychiatric disorder frequently identified in the perpetrators, parricides are often committed with undue violence and result in overkill [9, 16, 23]. This term refers to the infliction of massive injuries that far exceed what would be necessary to kill the victim [42], or to two or more separate actions of stabbing, cutting or shooting, or a severe beating involved in the process of slaying the victim [44]. As a matter of fact, the pattern of injuries inflicted on the victim in parental homicide is seldom described in the scientific articles regarding parricide. Some authors suggest that the killing of Dictator Julius Caesar in 44 B.C. could be considered the most ancient example of parricide resulting in overkill committed by his son and a group of conspirators, who struck him 23 times with daggers and continued stabbing him as he lay defenseless before the Senate [45]. One case of patricide perpetrated by shooting and stabbing [23], and one carried out by blunt force injuries to the victim’s head and multiple stabbing with scissors and a screwdriver on the torso [4] are described in the literature.

In the present case, the criminological analysis recalls several of the characteristics of adult parricide offenders discussed in the literature. The offender was a middle-aged, unmarried, unemployed man, living with his father. He also presented a long history of psychiatric disorders, having been diagnosed firstly with a bipolar disorder and personality disorder about 20 years before the murder. Despite his treatment adherence being reported as good for several years, there is evidence that the day before the murder he had refused treatment, and his father had requested an appointment for his son at the outpatient service of the Public Department of Mental Health. The crime took place before the psychiatrist had the possibility to reevaluate him. The analysis of the murderer’s personal case file at the Department of Mental Health, however, disclosed that in the last 3 years his psychiatric outpatient follow-up had been characterized by a decreased adherence.

This report adds to the few existing data highlighting a possible association between delusional misidentification syndromes and homicidal behavior [20, 22]. Delusional misidentification syndromes include a group of psychopathological conditions often associated with brain damage or psychiatric disorders where patients misidentify persons, objects or themselves due to the delusional belief that they have been replaced or transformed [46]. Hence, in our case, the murderer’s delusional conviction that his father had been replaced by an impostor, as well as the persecutory delusion toward the perceived impostor, can be framed within a delusional misidentification syndrome [47], such as Capgras syndrome. The weight and bizarre nature of his delusional ideation, the absence of a critical capacity and the considerable distortion of reality present at the crime time led to the conclusion of criminal irresponsibility made by the forensic psychiatric expert.

As regards the manner of killing, this murder featured overkill by multiple fatal means. The murder stages were reconstructed by the forensic pathologist through the autopsy, whose results are interpreted in the light of the crime scene findings. The victim was found supine on the kitchen floor, with his t-shirt raised up above his chest and showing no tearing on its anterior portion, and he was loosely holding a kitchen knife in his right hand; the blade was consistent with the seven wounds, detected in the epigastric region. The wounds were close to one another and of different depths, with a single lethal injury of the abdominal aorta. These elements led the pathologists to hypothesize that the stab wounds were inflicted one after another in a short time period, when the man was already lying on the floor. The presence of the contusive head trauma with multiple ecchymoses on the scalp and the presence of a wide lacerated wound on the right frontoparietal region, which was adjacent to a broken marble baseboard, suggested that the perpetrator gripped his father’s head and smashed it against the baseboard. This trauma produced a slight subarachnoid hemorrhage in the right temporal lobe, detected at autopsy.

The fingerlike imprints of blood on the man’s face caused the pathologists to suppose that, at least partly, the blunt head trauma occurred after the stabbing, whereas the bruises on the back of the torso and on the upper limbs could be caused by the perpetrator shoving the victim, who fell down. The most interesting finding was the presence of a set of five keys with a whistle-like keychain in the victim’s throat, detected at inspection of the victim’s oral cavity, associated with superficial blood infiltrates of the tongue, of the palatine region, which also presented a small laceration of the mucosa, and of the hypopharynx. According to these findings, along with the presence of a bilateral hemorrhagic infiltration of the masseter and pterygoid muscles, the pathologists hypothesized that the perpetrator forced the set of keys down his father’s throat when the victim was still alive and gasping. From the murderer’s confession, this behavior can be assumed to be the result of a bizarre attempt to silence his victim. According to autopsy findings, the cause of death was ruled to be hemorrhagic shock due to stab wounds with omentum, mesentery, and aortic injuries coupled with severe cranioencephalic blunt force injury and asphyxia.

In reconstructing the dynamic of the homicide, we hypothesized that the aggression started with an altercation and subsequently led to physical assault, causing the victim to fall on the floor. Then the murderer repeatedly stabbed his father with the bread knife, causing the abdominal lesions (including the fatal one). Thereafter he violently banged his father’s head against the baseboard causing the blunt head trauma. In the last stage of the murder, the perpetrator pushed the set of keys down inside the victim’s throat in order to silence him. We also assumed that the knife was placed by the assailant in the victim’s hand at the end of the murder, as the handle was abnormal and inappropriate for threatening or hitting.

As commonly described in the scientific literature, also in our case the injury location differed according to the weapon type [40, 44], the blunt injuries being predominantly on the head, and sharp force injuries on the torso. The peculiarity of our case is certainly the manner of asphyxiation. Homicidal asphyxia by choking is very rare and is most frequently observed in infanticides [48, 49]. In adults it is mainly reported as an attempt to silence a victim that does not die immediately and tries to call for [50]. Only two cases of homicidal choking in adults, using toilet paper, have been reported, involving a patient with schizophrenia hospitalized in a mental hospital and an old woman with Alzheimer’s disease [51, 52]. In all the cases described in the literature, the victims are choked/smothered using soft means like tissue paper, whereas in our case hard, sharp objects were forced down the victim’s throat, making this case exceedingly rare.

Existing data on homicides suggest that bodies found at the same scene where they are slain, facing up and uncovered, with multiple wounds to one body area, are usually victims of a homicidal impulsive behavior with no great degree of planning by the perpetrator [53]. Nonetheless, the homicide, committed by the use of multiple fatal means based on a delusional state with no consciousness alterations, and with an escalating outrage of violence, raises the possibility of attributing a fair degree of planning and finalization of the murder act, albeit in redundant and excessive ways.

## Conclusion

Parricide is a complex, behaviorally and criminologically heterogeneous crime, as it results from a multiplicity of personal, environmental and clinical factors. The case reported here highlights the association between a major psychiatric disorder (schizoaffective disorder), in a phase of acute decompensation with delusional misidentification syndrome, resulting in overkill which includes a rare form of asphyxiation. The evocative relationship between severe mental disorders, delusional misidentification syndrome and overkill deserves further attention.

## Keypoints

1. Parricide is a term used to identify offspring-perpetrated homicides. It is an infrequent event accounting for only 2–5% of all homicides.
2. Psychosis with delusion or hallucinations is regarded as the main motivating factor for adult-perpetrated parricides.
3. Delusional misidentification syndromes, including Capgras syndrome, are rare psychiatric conditions which can be associated with homicidal behavior.
4. Parricide is often committed with undue violence and may result in overkill. It is sometimes carried out using multiple fatal means.
5. Homicidal asphyxia by choking is a very rare occurrence and it seldom involves adult victims.
